# 92. Incidence of *Adenovirus* Respiratory Infection and Coinfection in a Longitudinal Birth Cohort

**DOI:** 10.1093/ofid/ofac492.017

**Published:** 2022-12-15

**Authors:** Adam E Gailani, Zheyi Teoh, Shannon C Conrey, Rachel M Burke, Allison R Cline, Marie E Killerby, Xiaoyan Lu, Claire Mattison, Monica McNeal, Ardythe L Morrow, Daniel C Payne, Mary A Staat

**Affiliations:** Cincinnati Children's Hospital Medical Center, Cincinnati, Ohio; Cincinnati Children's Hospital Medical Center, Cincinnati, Ohio; University of Cincinnati College of Medicine, Cincinnati, Ohio; CDC, Atlanta, Georgia; University of Cincinnati College of Medicine, Cincinnati, Ohio; CDC, Atlanta, Georgia; Centers for Disease Control and Prevention, Atalnta, Georgia; Centers for Disease Control, Atlanta, Georgia; Cincinnati Children's Hospital Medical Center, Cincinnati, Ohio; University of Cincinnati College of Medicine, Cincinnati, Ohio; US Centers for Disease Control and Prevention, Atlanta, Georgia; CCHMC, Cincinnati, Ohio

## Abstract

**Background:**

Adenoviruses (AdVs) are a common cause of acute respiratory illness (ARI) in children and are often detected with other viruses (coinfection). However, the community incidence of AdV infections is poorly understood due to a lack of prospective studies outside the hospital setting. Here, we aim to characterize respiratory AdV infections and coinfections in a prospective birth cohort of children.

**Methods:**

The PREVAIL cohort is a CDC-funded, 2-year birth cohort, conducted from 2017–2020 in Cincinnati, OH. ARI was defined as the presence of cough or fever identified with weekly maternal text surveys and medical chart review. Mid-turbinate nasal swabs were collected weekly. Swabs were tested using Luminex Respiratory Pathogen Panel. AdV infection was defined as a swab positive for AdV and included subsequent positives < 30 days apart. Coinfection was defined as detection of any other virus(es) during an AdV infection. Children who submitted at least 70% of weekly samples were included in our analysis.

**Results:**

101 children met inclusion criteria, representing 165 child-years. 137 distinct AdV infections were identified (incidence 0.84 infections per child year), with 98 (97%) children having ≥1 AdV infection. Only 40% (n=55) of AdV infections were symptomatic. Of those with symptomatic infections, 51% (n=28) sought medical care, with 42% (n=23) presenting to a primary care provider and 9% (n=5) resulting in an ED visit or hospital admission. Coinfections were detected in 67% (n=92) of AdV infections, with 45% (n=62) coinfected with 1 virus, 19% (n=26) with 2 viruses, and 3% (n=4) with ≥3 viruses. 77% of coinfections (n=71) were rhino/enterovirus. The number of coinfections or the specific coinfection virus was not associated with an increase in symptom prevalence or symptom severity (all p > 0.05).

Viral Coinfection Frequency with Adenovirus Infection in the PREVAIL Cohort.

Adenovirus Infection and Coinfection in the PREVAIL Cohort.

**Conclusion:**

In this cohort of healthy children, AdVs were a common cause of respiratory infection. Most infections were asymptomatic or resulted in mild symptoms. Two-thirds of AdV infections involved viral coinfections, but coinfection was not associated with more frequent or severe symptoms. Our findings suggest studies that only include symptomatic or hospitalized patients may overestimate AdVs disease severity.

**Disclosures:**

**All Authors**: No reported disclosures.

